# Cholera

**Published:** 1854-08

**Authors:** 


*Cholera.*—A cholera zone seems to be now fully established, extending nearly due west from Boston across the continent. As usual in the erratic invasions of this disease, some cities immediately on the line of the zone have escaped, while others are suffering to a considerable extent.

The epidemic of this year has encroached upon the fancied security of the granite regions, and many cases have occurred in Boston. We hear of a limited amount of disease in New York and Brooklyn. At Buffalo, the first case made its appearance on the 2d of June, but it has not as yet reached the condition of an epidemic. Its progress has been slow, and the average of success in treatment has been fair for the opening of the epidemic. Up to July 1st, there were but twelve cases, of which eight died. From the 1st of July to the 23d, inclusive, there were 126 cases with 66 deaths. Many of these had no medical aid. The deaths are confined, with three or four exceptions, to foreigners.

Since the 23d, there has been a considerable accession to the disease, and there is a general tendency to diarrhœa. But little is known as to the success of the various modes of treatment. But it is evident, that, taking into consideration all the circumstances, the relative mortality has not been great. As we mentioned before, quite a number of cases are found on the list of permits for burial, mentioned as having had no physician. Others have been treated under the most unfavorable circumstances.

We would mention that a large number of deaths occurred at the County Poor House, under circumstances which have roused a strong feeling of indignation in the community. We publish, on another page, an article from the Commercial Advertiser of the 20th, the authorship and responsibility of which we here assume.

It is also our duty to record a most fatal visitation of Cholera at Suspension Bridge, near Niagara Falls. Probably no locality in this country has ever suffered so severely, unless we except Sandusky. But it is doubtful whether even that epidemic, terrible as it was, equaled in fatality, and in the mournful and shocking character of its incidents, the one at Suspension Bridge. In the hurry of our present writing we have only time to mention a few of the more prominent features, reserving a full account for our leisure.

The disease was principally confined to the large foreign population engaged in the excavations and public works. It was characterized by a peculiar malignity, many cases living but six hours after the attack; while in numerous instances there was no premonitory diarrhœa. Some sixty deaths occurred during the first week. The panic was indescribable. Patients went into collapse, immediately after their attack, while some perished from sudden congestion.

It was our privilege to be associated with Dr. Rogers, the physician of the place, during three prolonged visits which we made. Prof. Hamilton also spent twenty-four hours on the spot, during the greatest malignity of the epidemic.

Treatment, in the majority of cases, availed but little. Nurses could not be procured upon the Canada side, and in our rounds on that shore we frequently found men left alone, with a bucket of water by their side, to die uncared for. Aside from the neglect of patients induced by the panic, other effects were evident in the desperation, frantic fear, or cold apathy and fatalism, manifested by many.

Dead bodies (four in number) were in one instance got rid of by burning the shanty in which they lay.

These remarks apply only to the Canada side. There the principal men—those who should have remained—fled, leaving their men to die unassisted. On the American side, the prominent citizens of the place remained, and were most efficient in sanitary measures. Col. Fiske, Mr. Adams, and others, evaded no responsibility; and as a consequence the fatality, though great, was not accompanied by scenes dishonorable to human nature.

When we left this morning, (26th July,) no new cases had occurred during the night on the American side. During the preceding twenty-four hours there were four deaths; three of them Americans. Several cases were then convalescent.

We have given this brief mention of this epidemic in the understanding that an article will be prepared by Prof. Hamilton, illustrating its causation and peculiar features: many of which possess unusual scientific interest.

